# Invasion by an ecosystem engineer changes biotic interactions between native and non‐native taxa

**DOI:** 10.1002/ece3.9820

**Published:** 2023-02-22

**Authors:** Anna J. Holmquist, Seira A. Adams, Rosemary G. Gillespie

**Affiliations:** ^1^ Department of Environmental Science, Policy and Management University of California: Berkeley Berkeley California USA

**Keywords:** biotic interactions, *Hedychium gardnerianum*, metabarcoding, *Pagiopalus*, species invasion

## Abstract

Earth systems are nearing a global tipping point, beyond which the dynamics of biological communities will become unstable. One major driver of instability is species invasion, especially by organisms that act as “ecosystem engineers” through their modification of abiotic and biotic factors. To understand how native organisms respond to modified habitat, it is essential to examine biological communities within invaded and non‐invaded habitat, identifying compositional shifts in native and non‐native taxa as well as measuring how modification by ecosystem engineers has affected interactions among community members. Using dietary metabarcoding, our study examines the response of a native Hawaiian generalist predator (Araneae: *Pagiopalus* spp.) to habitat modification by comparing biotic interactions across metapopulations of spiders collected in native forest and sites invaded by kāhili ginger. Our study shows that, although there are shared components of the dietary community, spiders in invaded habitat are eating a less consistent and more diverse diet consisting of more non‐native arthropods which are rarely or entirely undetected in spiders collected from native forest. Additionally, the frequency of novel interactions with parasites was significantly higher in invaded sites, reflected by the frequency and diversity of non‐native Hymenoptera parasites and entomopathogenic fungi. The study highlights the role of habitat modification driven by an invasive plant in altering community structure and biotic interactions, threatening the stability of the ecosystem through significant changes to the biotic community.

## Graphical abstract

Using dietary metabarcoding, our study examines the response of a native Hawaiian generalist predator to habitat modification by comparing biotic interactions across metapopulations of spiders collected in native forest and sites invaded by kahili ginger. Our study shows that, although there are shared components of the dietary community, spiders in invaded habitat are eating a less consistent and more diverse diet consisting of more non‐native arthropods which are rarely or entirely undetected in spiders collected from native forest. Additionally, the frequency of novel interactions with parasites was significantly higher in invaded sites.

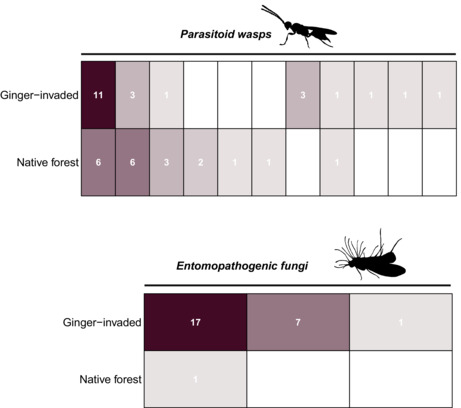

## 1 INTRODUCTION

Over recent decades, global human transportation networks have led to the establishment of once geographically restricted species into new ecosystems (Hulme, 2009; Hulme et al., 2008; Roderick & Navajas, 2015; Sinclair et al., 2020). While many introduced taxa go unnoticed, some may act as “ecosystem engineers” by altering abiotic and biotic factors, leading to changes in the structure of the original ecosystem (Jones et al., 1994); such non‐native taxa may then be called invasive because of their negative impact on ecosystem services and native species (Richardson et al., 2011). Invasive plants are particularly pervasive and act as ecosystem engineers by changing soil chemistry (Ehrenfeld, 2003), nutrient cycling (Weidenhamer & Callaway, 2010), microclimates (Ruckli et al., 2013), and the presence and abundance of native taxa (Schirmel et al., 2016). Because of their role as primary producers, the introduction of new plants and the resulting displacement of native flora can produce strong bottom‐up effects with far‐reaching consequences (Bezemer et al., 2014).

Native arthropods, and in particular herbivores, are often strongly affected by plant invasion due to their direct interaction with native plants. Non‐native flora can induce evolutionary traps for native insects by producing attractive habitat that in fact has fitness costs for species with particular physiological adaptations (Harvey & Fortuna, 2012; Keeler et al., 2006; Schlaepfer et al., 2005). Alternatively, native taxa with higher behavioral plasticity may be capable of rapid host shifts and benefit from the increased access to resources provided by new plant species (Keeler & Chew, 2008; Schlaepfer et al., 2005). The contrasting response of different species to plant invasion results in varied changes to the overall arthropod community. Most studies document decreases in richness and abundance levels in invaded patches but this result is inconsistent (Bezemer et al., 2014; Litt et al., 2014; Schirmel et al., 2016).

A major driver of abundance and/or richness changes are shifts in biotic interactions, such as increased predation, lack of the most nutritionally beneficial prey items, or a higher interaction with parasites (Mattos & Orrock, 2010; Suarez et al., 2000). The displacement of native taxa and associated changes in biotic interactions can result in invasional meltdown, in which modification driven by one invasive provides an opportunity for the establishment of other non‐native taxa (Green et al., 2011; Simberloff & Von Holle, 1999). The introduction of multiple non‐native taxa can result in a significant disruption to the native food web (Borges et al., 2006; Cucherousset et al., 2012; Wainright et al., 2021), causing broad ecosystem effects. Because of the widespread consequences that can occur following invasion, it is important to go beyond common diversity metrics such as abundance or species richness and study community network structure across multiple trophic levels (Harvey et al., 2010).

Kāhili ginger (*Hedychium gardnerianum*), a plant native to the Himalayas, has expanded its range globally to the Azores, Madeira, Jamaica, Réunion, New Zealand, and Hawaii, as well as across South and Central America, Australia, and Southern Africa (Pereira et al., 2021). *Hedychium gardnerianum* was brought to Hawaii in 1940 and has become aggressively invasive, capable of establishing in intact native rain forest, displacing understory vegetation, altering the composition of soil microbial decomposers, and promoting the establishment of other plant invasives (Minden et al., 2010; Santos et al., 1992; Vorsino et al., 2014). Here, we use a recent invasion by kāhili in Hawaii to measure how the establishment of an invasive ecosystem engineer changes biotic interactions. By assessing the major shifts that occur in biotic interactions following the introduction of ginger using the diet of a native generalist predator, we can ask if major shifts in biotic interactions occur following invasion. Toward understanding the effect of ginger invasion on the interactions between native and non‐native taxa, it is essential to identify a highly simplified system where ginger invasion is the primary differentiation between sites. Our sampling sites were located in the mesic forest of Waikamoi on East Maui, where a sharp boundary exists between ginger invasion and the native forest due to the efforts of the Nature Conservancy of Hawaii in protecting their lands (The Nature Conservancy of Hawaii, 2011). The adjacency of native forest and ginger‐invaded sites allows studying invasion in discrete units at a small spatial scale not usually possible in invasion biology.

To further reduce variability, we chose a single endemic genus of spider predators (*Pagiopalus*, Philodromidae) to assess biotic interactions (Suman, 1967). While little is known about the ecology of this endemic genus, species in the family Philodromidae are generalist active hunters found on foliage (Cardoso et al., 2011). Like most spiders, they are expected to consume prey at rates roughly proportional to their availability in the environment. Therefore, we use these spiders as a vehicle to study the relative proportions of native and non‐native species, as well as shifts in biotic interactions. We utilized metabarcoding to compare diet composition and parasite loads in spiders across adjacent sites in ginger‐invaded habitat and native forest. By assessing biotic interactions using the diet of a native generalist predator in a simplified system, we can ask if major shifts in the community network occur following invasion.

We have three hypotheses related to the effect of an invasive ecosystem engineer on relative proportions of native and non‐native taxa and the associated shifts in biotic interactions. First, the altered environmental conditions in ginger will result in arthropod communities differing from native forest sites; we expect to see this reflected in compositional differences in the diets between spiders collected in ginger‐invaded habitat and native forest. Second, ginger sites will host more non‐native prey items, reflected again in the diet of *Pagiopalus* specimens. Lastly, a higher interaction between arthropods and non‐native parasites will exist in invaded forest, due either to parasitism of the spider or by secondary consumption.

## 2 METHODS

### 2.1 Study system

The study site sits across two adjacent reserves on East Maui—The Nature Conservancy (TNC) of Hawaii's Waikamoi Preserve and the Makawao Forest Reserve. This area was invaded by ginger in the early 1980s. The ginger has spread across the reserves, significantly increasing in density and coverage over the last decades (The Nature Conservancy of Hawaii, 2011). The Waikamoi Preserve is actively managed by TNC, who regularly remove ginger seedlings throughout the preserve to maintain a largely native landscape within the fenced‐off area. In comparison, the Makawao Forest Reserve has maintained less frequently and thus a thick stand of ginger covers much of the reserve, meeting the fence line that separates the adjacent Waikamoi Preserve.

### 2.2 Sampling

**FIGURE 1:**
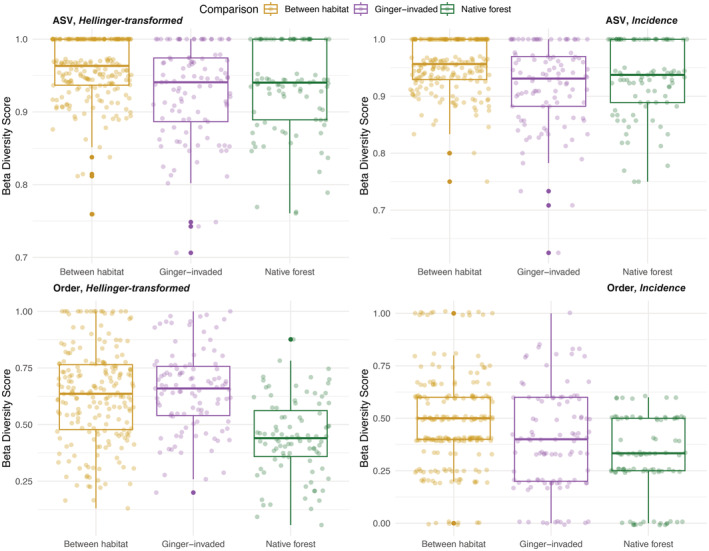
Dietary dissimilarities using beta diversity between invaded and native forest sites (yellow), within ginger‐invaded sites (purple) and within native forest sites (green). Column 1 shows beta diversity calculated using incidence data, based on ASVs (top) and order (bottom). Column 2 shows beta diversity calculated using Hellinger‐transformed reads, based on ASVs (top) and order (bottom).

To investigate the effects of invasive ginger, we laid five transects in the native mesic forest habitat of the Waikamoi Preserve and five transects in ginger‐invaded habitat of the Makawao Forest Reserve (Figure 1; see Data Accessibility for coordinates). Each transect ran 30 m long and was 3 m in width. Spiders were collected along transects between June 8 and June 21 in 2017 using vegetation beat sampling. Each transect was broken into three blocks, each 10 m in length. Combined transect number and block are used as “sampling units” throughout the paper for a total of 30 unique units. Vegetation along transects was sampled using a beat sheet. Fifteen areas of vegetation were beaten in each block of each transect, totaling 75 s of beating per block. Spiders were collected from the beat sheet using an insect aspirator and preserved in 100% EtOH in individual 2 mL vials. Samples were stored in a −20°C freezer until further use.

### 2.3 Molecular procedures

Spiders were identified morphologically and *Pagiopalus* specimens were retained. To extract DNA from the gut of each spider, the opisthosoma (abdomen) was first cleaned using 70% ethanol and water to remove external DNA contamination. The opisthosoma was removed using a sterile scalpel blade and placed in Qiagen cell lysis solution. The opisthosoma was then ground using two 3‐mm steel beads on a Genogrinder (Spex SamplePrep, Metuchen, NJ, USA) for 2 min at 1200 hz. DNA from ground samples was extracted using the Qiagen Puregene kit (Qiagen, Hilden, Germany) according to the manufacturer's protocol. DNA was then amplified using primer sets for 28 s, 18 s, and 16 s, optimized for spider gut content amplification (Krehenwinkel et al., 2019; Table S1). COI was unsuccessful at amplifying prey in this genus and was not used (Krehenwinkel et al., 2019). 28s has been used for fungal identification (Xu, 2016; Zhao et al., 2011) and was therefore appropriate for detecting both arthropods in spider gut content and fungi. Amplification was performed using the Qiagen multiplex PCR kit (Qiagen, Hilden, Germany) in 10 μL reactions with 1 μL of template DNA, and 1 μL of 10 μM primer dilutions of each primer for 35 PCR cycles. Nuclear markers were multiplexed and amplification was performed at an annealing temperature of 55°C. Amplification of the mitochondrial 16s was performed independently at an annealing temperature of 46°C. Negative PCR controls were included in each round of amplification. Primers included a 5′ tail that allowed binding of 8 bp indexing primers and TruSeq adapters (Illumina San Diego, CA, USA) performed in a second indexing PCR of 5 cycles (Krehenwinkel et al., 2019). PCR products were visualized using a 1.5% agarose gel. Products were pooled in approximately equal amounts based on band strength. 1X AmpureBeads were used to clean the pooled products. Negative controls were included in the final library. The cleaned library was then sequenced on an Illumina Miseq using V3 chemistry alongside other libraries not associated with the project; 1.71 million reads were expected.

### 2.4 Bioinformatics

Sequences were demultiplexed using Illumina Basespace (Illumina, San Diego, CA, USA). Demultiplexed sequences were batch processed using CutAdapt to remove primer sequences and perform preliminary quality filtering (Martin, 2011). The denoising algorithm DADA2 was run in R to produce amplicon sequencing variants (ASVs) and remove chimeras (Callahan et al., 2016). Parameterization and length trimming was dependent on the locus. Widespread contaminants were identified using package *decontam* in R using sequences identified in controls (Davis et al., 2018); the threshold argument was set to 0.5, which removes sequences more prevalent in controls than in true samples. Once contaminants were removed, ASVs were further curated using the LULU algorithm in R, which identifies NuMTs and any remaining artifactual sequences (Frøslev et al., 2017). Finally, sequences were filtered at the ASV and sample level by read counts: ASVs represented by <10 reads were removed and the number of reads found for an ASV within a sample that represented <0.01% of the total ASV reads or that represented <1% of the total reads for the sample were removed. Additionally, one sample had missing metadata and was removed.

ASVs were then written to FASTA files and using Geneious, assigned taxonomic identities through megablast. Full taxonomic lineage was assigned using a custom R script based on the package *rentrez* (Winter, 2017). Sequences were retained if the similarity was equal to or greater than 85% (Krehenwinkel et al., 2019). A species‐level identification was retained if the percent match was >=99%. There is little information on appropriate cut‐offs for assignment to genus or family using rRNA genes; 97% was used for genus and 95% was used for the family to avoid retention of incorrect taxonomic IDs. Only order was assigned if percent matches were lower (85–94%). To construct the prey data set, the phylum Arthropoda was selected from filtered sequences. Arthropod reads matching order Araneae were filtered to remove any sequences generated from the spider itself; while Araneae could include spiders eaten as prey, this was not confidently identifiable. Sequences in the order Hymenoptera were additionally removed from the prey dataset and treated as parasites because generalist spiders are rarely found to prey on Hymenoptera. Parasitism of Hawaiian Philodromidae is common (Suman, 1967) as is parasitism of their arthropod prey, making reads likely either secondary consumption or parasitism of the spider itself rather than prey reads.

Prey reads were assigned native or non‐native status based on publicly available literature. Likewise, sequences identified as entomopathogenic fungi and parasitic wasps were assigned to native versus non‐native status using publicly available literature. An order‐level network grid was constructed to visualize differences in ginger‐invaded sites and native forest using the package *bipartite* (Dormann et al., 2008). A network grid was also constructed to visualize the presence of different families of parasites in ginger‐invaded sites and native forest.

### 2.5 Statistical analyses

Community matrices were constructed using data generated from all markers. Both ASV and order were used as taxonomic units; order‐level matrices provided the coarsest view of diets while ASVs provided a species‐level view. Matrices were populated with either binary incidence data or Hellinger‐transformed relative read abundances. While read abundances have not been found to be true reflections of abundances, relative read abundances can provide useful information in analyses because presence–absence data places a higher emphasis on rare taxa and can increase the strength of compositional differences among communities (Laporte et al., 2021).

Hill numbers (Alberdi & Gilbert, 2019) were calculated to quantify prey diversity for individual spiders using *vegan* (Oksanen et al., 2013). Welch's t‐test was used to test the hypothesis of no differences in dietary prey diversity between spiders in ginger and native forest. Compositional differences were first assessed using taxonomic beta diversity, using package *BAT* (Cardoso et al., 2015). Because of the low dietary overlap between any two individual spiders discovered using beta diversity (Figure S2), sampling units were used for further compositional analyses. Beta diversity values were calculated between sampling units using package *BAT*. Differences across sites grouped between habitat and within each habitat were tested using ANOVA and Tukey's test was used to test if sites between habitats were more compositionally dissimilar than sites within either ginger‐invaded or native forest sites. To assess overall differences between the diets of spiders in ginger sites versus native forests, distance matrices were calculated using Jaccard distance for incidence data and Hellinger distance for relative read abundances. Non‐metric dimensional scaling (NMDS) was performed using each distance matrix using a maximum of 1000 random starts; *k* = 3 was used for ASV matrices and *k* = 2 for order matrices to achieve convergence. To test for group differences, permutational multivariate analysis of variance (PERMANOVA) was performed with ginger‐invaded and native forest as the independent grouping variables using *vegan*. Multivariate homogeneity of group dispersions was tested using package *vegan*.

Parasite frequency was calculated using the number of ASVs per spider within ginger and native forest sites that were identified as parasitic. Native and non‐native status as well as most common arthropod host was determined by the literature for taxa identifiable to genus or species. Welch's t‐test was used to test the hypothesis of no differences in parasitic load between ginger and native forest sites.

Analyses were conducted in R version 4.2.2 and Geneious Prime v. 2022.0.2. Data and code for analysis are available on GitHub and Dryad (see Data Availability Statement).

## 3 RESULTS

### 3.1 Summary of collections

A total of 168 *Pagiopalus* spp. were collected in total; abundances were nearly equal across ginger and native forest with 82 specimens collected from ginger sites and 86 specimens collected from native forest. There were, on average, 5.79 ± 0.55 spiders collected and 16.8 ± 2.4 spiders collected in total per transect.

### 3.2 Summary of molecular findings

Of the 1,370,873 reads retained following DADA2 and additional filtering, 1,059,644 reads were not prey reads; this reduction came largely from sequences of the spiders themselves (679,534 or 64.1% Philodromidae reads) followed by fungi (234,630 reads, 22.1%) (Figure S3). Sequences of spiders were almost exclusively produced by the 28s marker pair (679,127 of 679,534 reads). Order Hymenoptera represented an additional 60,060 (5.7%) of the non‐prey reads. Spiders collected in ginger‐invaded habitat produced more reads than spiders collected in native forest prior to filtering (1,111,019 reads versus 262,213 reads). This came from the presence of additional phyla and non‐arthropod reads in higher abundances, particularly Basidiomycota which returned 136,932 reads from ginger and only 35,212 from native forest. Additionally, there were more reads identified as the spiders themselves in the samples from the ginger‐invaded sites (518,456 vs. 161,078), which may indicate higher sequencing coverage for unidentifiable reasons.

Following prey read curation, a total of 311,229 reads were retained from 82 spiders collected in ginger‐invaded sites and 63 spiders collected in native forest for a total of 145 spiders. There was an average of 3468.9 ± 449.0 reads from the spiders in invaded habitat and 700.8 ± 133.2 from spiders in the native forest transects, reflecting the higher sequencing depth for the spiders in the ginger‐invaded sites. 16s returned the highest number of prey reads (205,236 reads) and ASVs (110 ASVs), followed by 28s (91,148 reads and 44 ASVs) and lastly 18s (14,845 reads and 10 ASVs). This was reflected in the sequencing success across samples; prey reads were produced for 112 specimens using 16s, 76 specimens using 28s and 17 specimens using 18s reads.

### 3.3 Summary of taxonomy

There were 23 species, 32 genera, and 31 families belonging to nine orders detected from the gut content of the *Pagiopalus* across ginger and native forest sites (Table S2). Only 23.2% and 37.8% of ASVs returned a confident species or genus ID, respectively. This was improved at the family level; 61.6% of ASVs returned family IDs at a 95% match. 16s sequences returned the lowest matches at 91.58% pairwise identity on average. While returning the lowest ASVs and retained specimens, 18s had the highest matches at 98.27%; 28s was near with an average match of 97.72%. The low percent identity matches for many ASVs biased taxonomic results at the species and genus level toward well‐known taxa present in GenBank.

### 3.4 Prey diversity and abundance

Of the total 164 ASVs in the dataset, only 29 ASVs were shared across ginger and native‐forest sites. Dietary richness was higher in ginger‐invaded habitat than in native forest, with 66 total ASVs found in native forest compared to 127 total ASVs found in the spider diets in ginger‐invaded sites. Taxonomic composition showed similar trends; 11 of 31 families, 10 of 32 genera, and 5 of 23 species were shared across ginger and native forest sites.

Hill numbers were used to quantify prey diversity between ginger‐invaded and native forest spiders based on ASV and taxonomic identity. Spiders rather than sites were used to examine dietary breadth at the individual level. Mean values for dietary richness were determined to be significantly different between ginger‐invaded habitat and native forest for both ASV and order (*p*‐value <.01; Table 1), with spiders in ginger sites having higher diversity on average, representing a wider niche breadth than spiders in native forest.

**TABLE 1:** Results of the Welch's *t*‐test for differences in Hill numbers (*q* = 0, 1, 2) between invaded and native habitat using ASV or order diversity. Values were significantly different at all values and taxonomic levels.

	*q*	Mean difference	Ginger invaded	Native forest	*t*‐statistic	*p*‐value
ASV	*q* = 0	0.782	2.885	2.103	3.253	.0014**
ASV	*q* = 1	0.656	2.522	1.866	3.33	.0011**
ASV	*q* = 2	0.575	2.309	1.734	3.352	.001**
Order	*q* = 0	0.432	1.846	1.414	3.635	4e‐04***
Order	*q* = 1	0.341	1.667	1.326	3.381	9e‐04***
Order	*q* = 2	0.298	1.578	1.28	3.25	.0015**

*Note:* **p < .01, ***p < .001.

### 3.5 Dietary composition

Beta‐diversity values based on individual spider comparisons were dominated by values of 1 (entirely dissimilar diets) (Figure S2). Because of this high dissimilarity, sampling units were used to further explore dietary differences between ginger and native forest; this was done to allow assessment of the differences between habitat, rather than the dietary diversity within *Pagiopalus*. For both order‐ and ASV‐data, there were significant differences in compositional dissimilarity between groups—comparing diets within invaded habitat, diets within native forest, and diets between invaded and native forest (Figure 1). Using ASV data, the dissimilarity values between the diets in ginger‐invaded habitat and native forest were significantly different from diets within either habitat type using both ASVs and order (*p*‐value <.001). However, beta diversity was generally high using ASVs (x̅_H_ = 0.944, x̅_I_ = 0.940). Different trends emerged when using order; in particular, a higher dissimilarity of diets within ginger itself was detected. When using incidence data, the dissimilarity of diets within ginger was in fact higher (x̅_I_ = 0.650) than the dissimilarity of the diets across invaded and native habitat (x̅_I_ = 0.629). Diets within native forest were the most similar (x̅_H_ = 0.444, x̅_I_ = 0.311).

**FIGURE 2:**
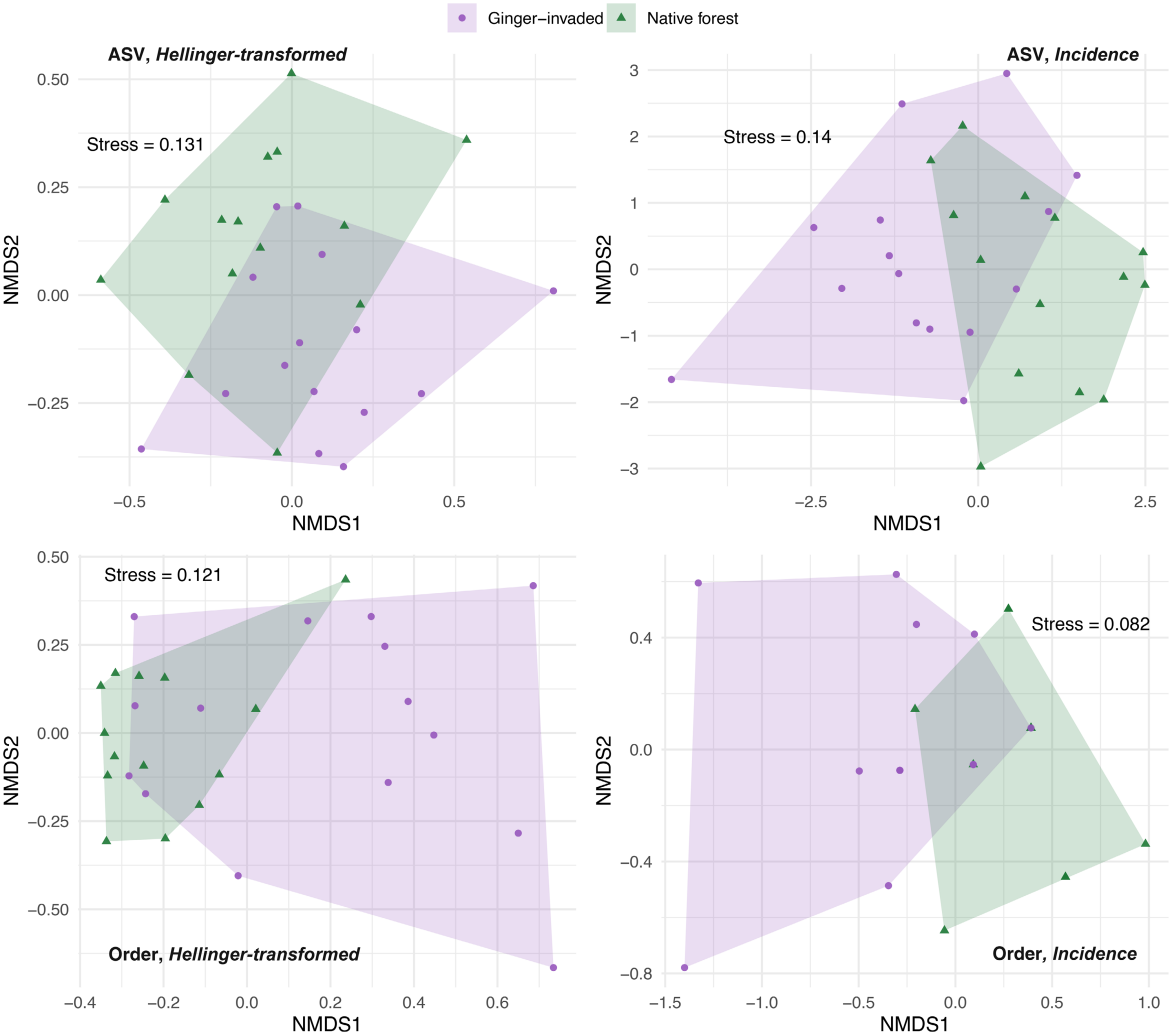
NMDS plots of dietary composition at each site, with shape and color representing sites in ginger‐invaded habitat (purple points) and native forest (green triangles). Column 1 shows distances calculated using incidence data, based on ASVs (top) and order (bottom). Column 2 shows distances calculated using Hellinger‐transformed reads, based on ASVs (top) and order (bottom).

No significant difference in group dispersions was detected for any matrix combination using PERMDISP2 (Table S3). When performing PERMANOVA using Hellinger distance, no significant differences emerged between the dietary composition of ginger‐invaded sites and native forest, using ASV or order (Table 2). However, when using incidence data, significant differences were detected (Table 2) with the highest R^2^ value emerging when assessed by order. Visual assessment using ordination plots shows the higher overlap that is produced when using relative read abundances, while more separation exists when using incidence data (Figure 2).

**TABLE 2:** Results of PERMANOVA testing for group differences in dietary composition between spiders collected from invaded and native habitat. Distances calculated using incidence data show significant differences.

Level	Abundance type	*F*	*R* ^2^	Pr(>*F*)
ASV	Hellinger‐transformed	1.1785	0.0418	0.178
ASV	Incidence	1.2943	0.0457	0.041*
Order	Hellinger‐transformed	1.4396	0.0506	0.222
Order	Incidence	3.1205	0.1036	0.02*

*Note:* *p < .05.

**FIGURE 3:**
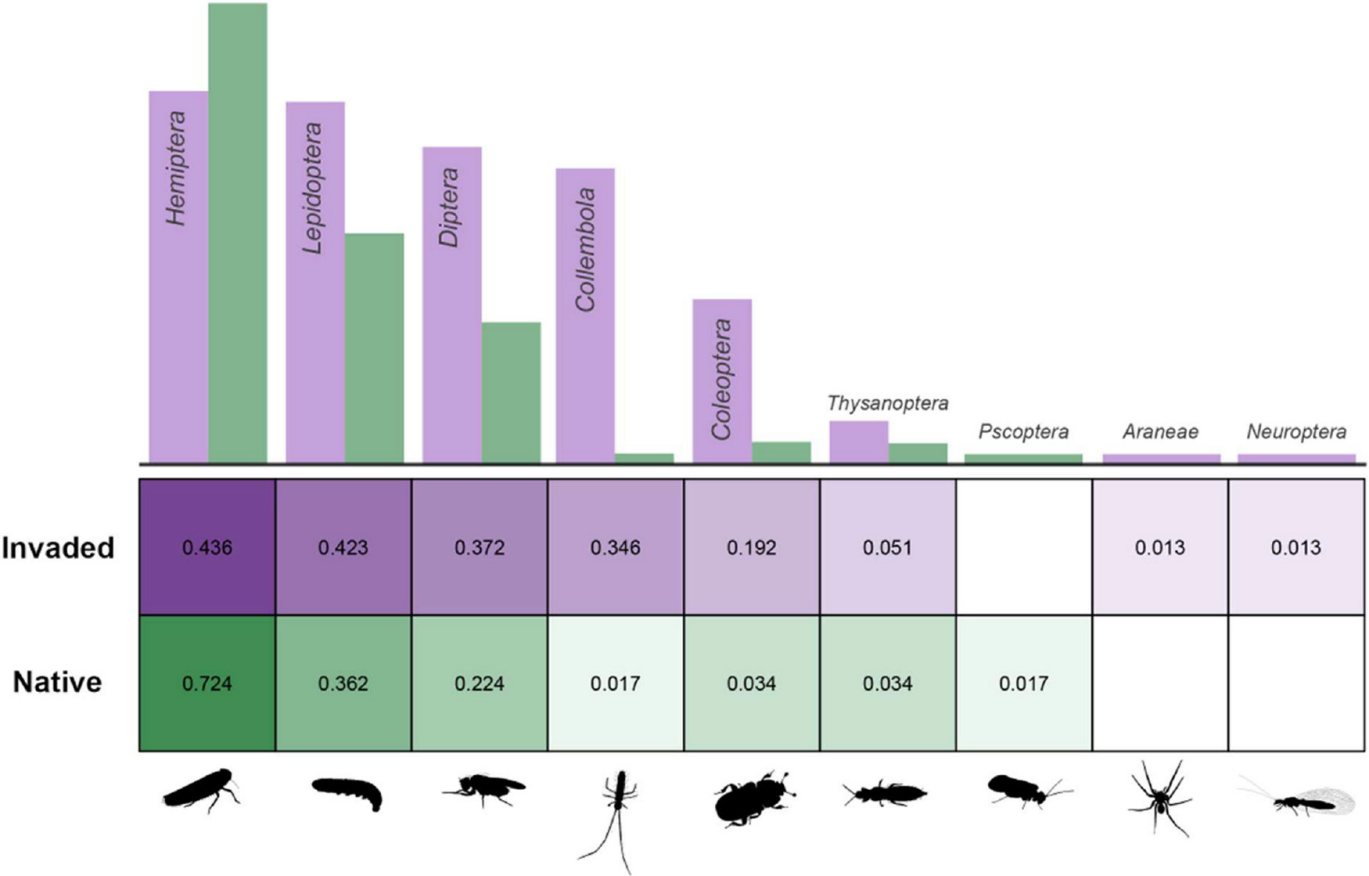
Proportion of spiders in ginger‐invaded habitat and in native forest eating different orders of arthropod prey.

There is high compositional turnover in the diets of spiders within the ginger habitat when we look at order‐level diversity; this is reflected in the beta‐diversity values (Figure 1) and in the NMDS plots (Figure 2), where the polygon encompassing ginger‐invaded sites occupies a larger portion of the ordination space. The diets of spiders in ginger‐invaded habitat are not dominated by any one order, with the most common prey (Hemiptera) detected in the diet of 43.6% of ginger spiders followed by Lepidoptera in 42.3% of spiders and Diptera in 37.1% of spiders. In contrast, spiders in the native forest are consuming more similar diets consisting predominantly of Hemiptera, with 72.4% of spider diets containing Hemiptera. The second most common order, Lepidoptera, only occurs in 36.2% of spider diets. Entomobryomorpha was the fourth most common prey group in ginger‐invaded habitat, detected in 27 spiders (34.6%) while Entomobryomorpha was only detected in one spider (1.7%) in native forest sites (Figure 3).

### 3.6 Native versus introduced prey items

**FIGURE 4:**
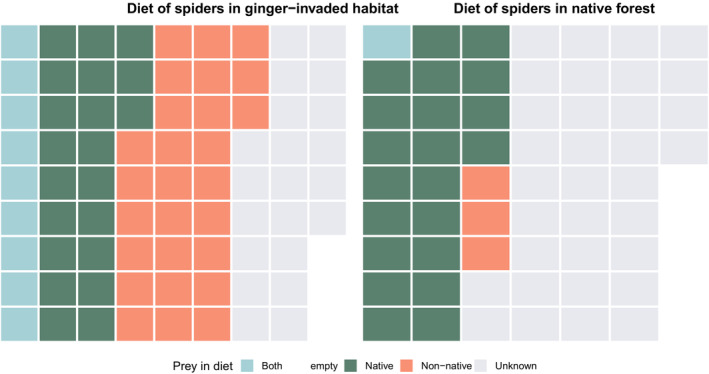
Dietary composition of each spider (represented by a single square), colored by whether prey in diet was entirely native, entirely non‐native, or mixed in origins. Grey squares represent diets consisting of unidentifiable prey items.

Because of low BLAST matches, only 53 of 164 ASVs were identifiable as native versus non‐native prey taxa. Therefore, most ASVs in the diets of spiders in ginger‐invaded or native forest were not assigned an endemism status. With those that were identifiable, we find more non‐native prey ASVs (20 ASVs) in ginger forest than in native forest (4 ASVs). Looking at the diets of individual spiders, we find that more spiders in ginger‐invaded habitat are consuming diets consisting entirely of non‐native prey (27 spiders) than diets consisting of native prey (21 spiders). Nine spiders were consuming both native and non‐native prey (Figure 4). In native forest, three spiders were detected eating entirely non‐native prey with an additional spider eating both native and non‐native. All Entomobryomorpha detected in the diets of spiders are non‐native. More unidentifiable sequences were detected in the native forest; the native/non‐native status in the diets of 33 of the 58 spiders from native forest was unknown while 21 of 78 spiders from ginger‐invaded forest had unknown diets. This could relate to the lack of presence in GenBank and indicate a higher concentration of native prey taxa, while adventive or introduced taxa are more well represented resulting in a higher level of ASV identification in ginger sites.

### 3.7 Parasitism

**FIGURE 5:**
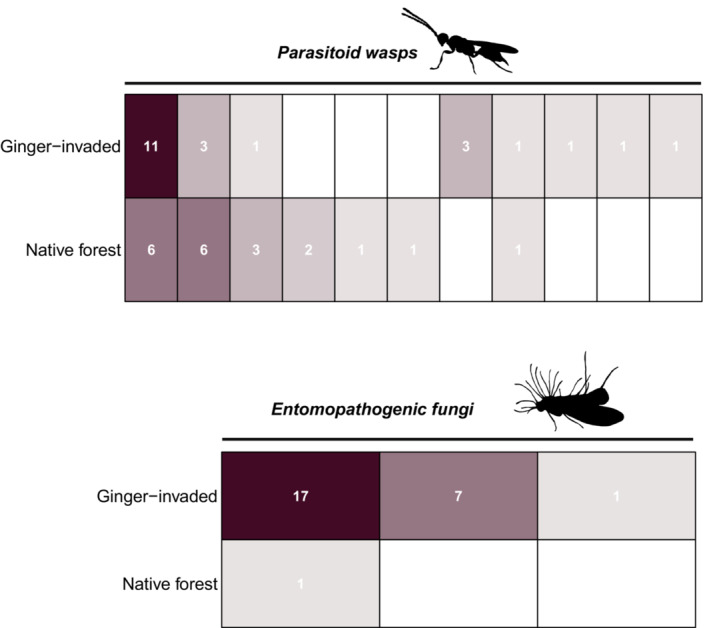
Number of spiders (in grid cells) detected with parasitoid wasp sequences and/or entomopathogenic fungal sequences in ginger‐invaded habitat and native forest habitat. Color intensity relates to the number of spiders.

Forty‐nine spiders had reads from entomopathogenic fungi or parasitic wasps (Figure 5). Using a number of ASVs associated with parasites in each individual spider, there was a slightly significant difference between ginger‐invaded habitat and native forest sites, with spiders from ginger‐invaded sites having an average of 0.58 ASVs associated with parasites compared to spiders from native forest having on average 0.35 ASVs associated with parasites (Welch t‐test; *t* = −2.07, *p*‐value = .0455). Eleven spiders collected from ginger‐invaded habitat had more than one ASV identified as parasitic compared to four from the native forest.

Thirty four of the 49 spiders had sequences identified as hymenopteran. Of the identifiable species, all but one were identified as non‐native (97.7% non‐native). The same number of spiders in ginger‐invaded habitat and native forest had parasitic wasp ASVs. It is worth noting that 18 spiders from ginger sites returned hymenopteran ASVs, in particular the parasitic Pteromalidae, but ASVs were removed due to BLAST matches under 85%; this reduced the number of spiders with parasitoids from 38 to 18 in ginger‐invaded habitat, while only removing one record of parasitized spider from native habitat.

Braconid wasps, predominantly *Rhopalophorus* and *Cotesia*, were most common among sites, although detected in a higher number of spiders from ginger‐invaded habitat (11) than native forest (6). This was followed by ichneumonid wasps, predominantly *Ichneumon*. Ichneumonid wasps were more common in native forest, detected in six spiders, than in ginger‐invaded habitat, detected in three spiders. Two genera of ichneumonids were unique to native forest. Families Bethylidae, Eucharitidae, Eulophidae, and Halictidae were unique to ginger‐invaded habitat, with Eucharitidae detected in three spiders. Families Diapriidae, Pteromalidae, Signiphoridae, and Sphecidae were detected only in native forest.

Entomopathogenic fungi were identified in 23 spiders in ginger sites and only one spider in the native forest, a difference that was highly significant using a number of ASVs per spider (Welch t‐test; *t* = −4.9051, *p*‐value = 4.411 e‐06). *Beauveria* was identified in 16 spiders in ginger, followed by *Ophiocordyceps* in seven spiders. Most spiders (21 of 23) had a single fungal type. However, *Beauveria* and *Ophiocordyceps* were found co‐occurring in two spiders in ginger forest. The single spider in native forest was identified with *Gibellula*, a known arachnid parasitic fungus. This genus was not detected in ginger sites, although it falls in the same family as *Beauveria*.

## 4 DISCUSSION

The goal of our study was to examine if invasion by a plant that drastically alters the environment would alter the relative composition of native and non‐native arthropod taxa and their associated biotic interactions. Using *Pagiopalus* spiders as a means for sampling arthropods to reveal both dietary composition and the presence of parasites, our results show major shifts in biotic interactions across native and invaded forest as well as an increased prevalence of non‐native prey taxa in invaded habitat.

Despite the different habitat produced by ginger, we found *Pagiopalus* in nearly identical abundances. Previous findings showed that the invasion of guava (*Psidium* spp.) in lowland forest of Hawaii is associated with an almost entirely endemic spider community (Gillespie, 1991; Gillespie et al., 1998) suggesting that vegetation does not constitute a major perturbation to generalist arthropods (Gillespie, 1999). Similarly, native Hawaiian land snails have been found to prefer invasive ginger to native plant species suggesting no apparent negative effect when the understory plant assemblage shifts (Meyer, 2012). However, as discussed earlier, diversity metrics such as abundance are inadequate to assess the true impact of invasion on native taxa; an equal abundance of *Pagiopalus* in ginger does not eliminate the possibility that invasion produces compositional shifts and changes biotic interactions.

Our results showed that, in areas modified by invasive ginger, spiders are consuming a partially overlapping but distinct spectrum of prey items compared to spiders in native forest (Figure 2). Our results showed that native taxa were found in the diet of the endemic *Pagiopalus* in both native forest and ginger‐invaded sites: *Campsicnemus* (Dolichopodidae) in 10 spiders from ginger sites and three from native forest, *Limonia* (Limoniidae) in 10 spiders from ginger sites and four from native forest. Hemipterans were detected in 42 of 58 spiders in native forest (72.4%), with the endemic genus *Nesophrosyne* (Hemiptera: Cicadellidae) detected in 13 spiders; hemipterans were also a common prey source in the spiders from invaded habitat but were detected in only 34 of 78 spiders (43.6%). Like some other hemipteran families, the family Cicadellidae is specific to native plant species (G. M. Bennett, pers comm, Bennett & O'Grady, 2012). As the dominant order in the diets of spiders collected in native forest and as herbivores which are often tightly associated with native flora, these hemipterans likely represent an important prey source with which *Pagiopalus* spp. evolved. Because the host plants are absent from heavily invaded ginger sites, access to host‐specific native prey may be limited for spiders in ginger‐invaded forest, and only accessible in sites in close proximity to native habitat.

The lack of native prey taxa may be connected to the varied diets found in the spiders from ginger‐invaded habitat which consisted of prey orders uncommonly found in the diets of spiders collected from native forest (Figure 3). In invaded sites, a more diverse prey community was detected in totality, reflected in the individual diets of spiders which showed wider breadth than spiders from native sites. Trophic dispersion has been detected in other studies following the invasion, which can be followed by destabilization of the food network and significant alteration to the biotic community (Wainright et al., 2021). The increased dietary diversity and the unique taxa being consumed in ginger‐invaded habitat point to changes in the broader arthropod community, although more general sampling would be needed to detect the extent of this change. Insertion of new interaction pathways may lead to destabilization and result in ecological state changes.

Differences between the diets of *Pagiopalus* in ginger and native‐forest sites were partially driven by the presence of non‐native taxa (Figure 4), specifically Entomobryomorpha (Collembola) which was detected in the diets of 28 spiders from ginger while being almost entirely absent in the diets of spiders from the native sites (Figure 3). The collembolan *Salina celebensis* was the most prevalent in ginger (detected in 18 of 82 spiders in ginger‐invaded habitat or 22.0%) and was not detected in native forest. This species, introduced from Asia, is characteristic of moist understory vegetation, with its extraordinary abundance noted in previous studies (Christiansen & Bellinger, 1994; Gruner et al., 2005; Gruner & Taylor, 2006). The other species of Collembola was *Tomocerus sp*; although not identifiable to species, *T. minor*, introduced from Europe has been known from Hawaii for >50 years (Christiansen & Bellinger, 1992). *Tomocerus* was found in a single spider in native forest in contrast to 15 spiders from ginger sites.

The much higher numbers of Collembola in invaded habitat may enhance the survival of native spiders such as *Pagiopalus*, simply because of their abundance; alternatively, they may serve to detract from their survival if they do not support the nutritional needs of the predator. Generalist predators are well documented to be dietarily selective when given a diverse set of prey, preferentially eating prey items that have the highest nutritional benefit (Michalko et al., 2019; Rendon et al., 2019; Toft, 1999). To obtain nutritional balance, however, a mixed diet consisting of nutritionally imbalanced or even toxic prey may be consumed. Previous work has shown that, while being a common source of prey in cursorial spiders (Birkhofer & Wolters, 2012), Collembola have mixed nutritional benefits. Studies have found the inclusion of certain species of collembolans (*Tomocerus bidentatus*, and *Isotoma anglicana*) in the diets of spiders increases reproductive output or survival while other species (*Folsomia candida*, *Folsomia fimetaria*, and *Isotoma trispinata*) drastically decrease reproductive output or result in high mortality (Møller Marcussen et al., 1999; Rickers et al., 2006; Toft & Wise, 1999). These studies additionally note the possible toxicity of certain collembolans, resulting in a significant fitness cost (Toft & Wise, 1999). Supplementation of Collembola and other prey not found in native forest may be a necessity for spiders in ginger‐invaded sites because of the lack of abundant host‐plant‐specific taxa; this could impart negative fitness costs. Further studies examining demographic structure of *Pagiopalus* spp. and quantifying the nutritional quality of common non‐native prey items would be necessary to determine the effect.

Parasitism is one fitness cost that is identifiable using high‐throughput sequencing approaches (Traugott & Symondson, 2008). Entomopathogenic fungi and hymenopteran taxa were detected in spiders collected from both ginger and native forest (Figure 5). We identified some well‐known lepidopteran parasitoids that have been accidentally or purposefully introduced, including *Cotesia vestalis*, *Ichneumon xanthorius*, *Meteorus laphygmae*, and the scale parasite *Aphytis chrysomphali*; these parasitoids are found in both native forest and ginger‐invaded sites. The major hymenopteran parasitoids were braconids, in particular *Microctonus* (*Rhopalophorus*) which is a well‐known adventive species across the islands (Nishida, 2002) and appears to parasitize beetles, notably chrysomelids (Beardsley, 1961). A hymenopteran ASV detected in a single spider belonged to family Halticidae, the sweat bees, while all other families and genera were confirmed to be parasitic.

Infiltration of native forest by non‐native parasitoids, in particular parasitoids of Lepidoptera, has been well documented (Henneman & Memmott, 2001). Their prevalence in spiders from native forest, then, is not surprising. We do find a differing composition of parasitoid wasps in ginger‐invaded habitat versus native forest as well as increased parasite co‐occurrence in spiders from ginger‐invaded habitat. Additionally, the choice of percent match threshold altered our results. At an 80% threshold, 32 spiders in ginger‐invaded habitat were detected with hymenopteran reads compared to 18 in native forest. At an 85% threshold, this was reduced to 17 spiders in ginger‐invaded habitat and 17 spiders in native forest. This clearly demonstrates the influence of filtering decisions on the results of a study.

Entomopathogenic fungal reads were detected predominantly using 28s; while not the most widely used marker for fungal DNA barcoding, 28s has been shown to be relatively effective (Xu, 2016; Zhao et al., 2011). For the sequences detected in the spiders in our study, their high percent identity matches and known presence in Hawaii provides more support for our finding. All ASVs identified as entomopathogenic fungi produced matches above 95% and, therefore, the number of spiders found with fungi did not change as was observed in hymenopterans. Entomopathogenic fungi were detected in 22 spiders in ginger sites while only found in one spider from native forest. This result is consistent with previous work in New Zealand which has shown that fungivores are much more abundant in sites that have been invaded by ginger (Bassett, 2014). The entomopathogenic fungi *Gibellula*, the most common spider fungal pathogen (Shrestha et al., 2019), was detected in only one spider in native forest. *Beauveria* was the most commonly detected fungus in spiders from ginger‐invaded sites, followed by *Ophiocordyceps*, a known parasite of beetle larvae (Wang et al., 2021). *Beauveria* is a genus of cosmopolitan fungal pathogens, associated with arthropods and the surrounding habitat, including in the soil and on vegetation. It has a wide host range of over 17 arthropod orders which includes spiders (Shrestha et al., 2019; Zimmermann, 2007), although infections have seldom been documented (Meyling & Eilenberg, 2007). In Hawaii, *B. bassiana* is used as a component of integrated pest management strategies in coffee plantations to control the coffee berry borer (*Hypothenemus hampei*), a major pest in Hawaii since 2014 (Greco et al., 2018; Hollingsworth et al., 2020). Multiple indigenous strains of *B. bassiana* are now found in Hawaii and detected in crops where there was no previous mycoinsecticide treatment (Castrillo et al., 2020; Hollingsworth et al., 2011).

While we cannot comment on the strain or arthropod host with which *Beauveria* was carried, its prevalence, along with the presence of other parasites, demonstrates a higher parasitic load found in the arthropods from ginger‐invaded sites. Parasitism of prey can impart indirect effects on predators by altering prey density or prey behavior. Spiders could benefit from the secondary consumption of parasites by increasing the nutritional gains from a single prey item. While we cannot make any conclusions about the effect of higher parasitism on the spiders themselves, the higher detection of parasites in ginger‐invaded habitat does demonstrate a cost for native arthropods, introducing new biotic interactions with potentially harmful taxa.

## 5 CONCLUSION

The combination of major dietary shifts driven by non‐native taxa and the high prevalence of parasite reads from spiders in ginger sites indicates a prominent effect of a plant invasion on the relative proportion of non‐native to native arthropods and the associated biotic interactions. The high density of non‐native taxa and increases in both parasitoid wasps and entomopathogenic fungi, as indicated through the vehicle of the *Pagiopalus* spider gut, clearly demonstrate that the sites modified by plant invasion are associated with a transformation of the arthropod community. The importance of this work is in highlighting how entire communities and the associated interactions are modified by a single invasive species that modify the environment. Cascading effects of ecosystem alteration and the restructuring of biotic interactions may contribute to extinction debt in invaded systems, where the full consequences of invasion do not become evident for many years (Kuussaari et al., 2009).

## AUTHOR CONTRIBUTIONS

**Anna J. Holmquist:** Data curation (lead); formal analysis (lead); visualization (lead); writing – original draft (lead); writing – review and editing (lead). **Seira A. Adams:** Conceptualization (lead); funding acquisition (supporting); methodology (lead); project administration (supporting); writing – review and editing (supporting). **Rosemary G. Gillespie:** Conceptualization (supporting); funding acquisition (lead); investigation (supporting); methodology (supporting); project administration (supporting); supervision (lead); writing – original draft (supporting); writing – review and editing (supporting).

## BENEFIT‐SHARING STATEMENT

Benefits from this research include accessible data and code as outlined above.

## Supporting information


Figure S1.

## Data Availability

The data and code used to perform analyses are publicly available on GitHub at ajholmqu/pagiopalus‐ginger. This includes (a) FASTA files containing all sequences, (b) CSV files with associated metadata, and (c) reproducible code to generate analyses.
