# Supplementary material for: Invasion by an ecosystem engineer changes biotic interactions between native and non‐native taxa
Source: Ecol Evol. 2023 Feb 22;13(2):e9820. doi: 10.1002/ece3.9820 (PMC9943940; doi:10.1002/ece3.9820)
Supplement: Supplementary file 1 — Figure S1. [file ECE3-13-e9820-s001.pdf]

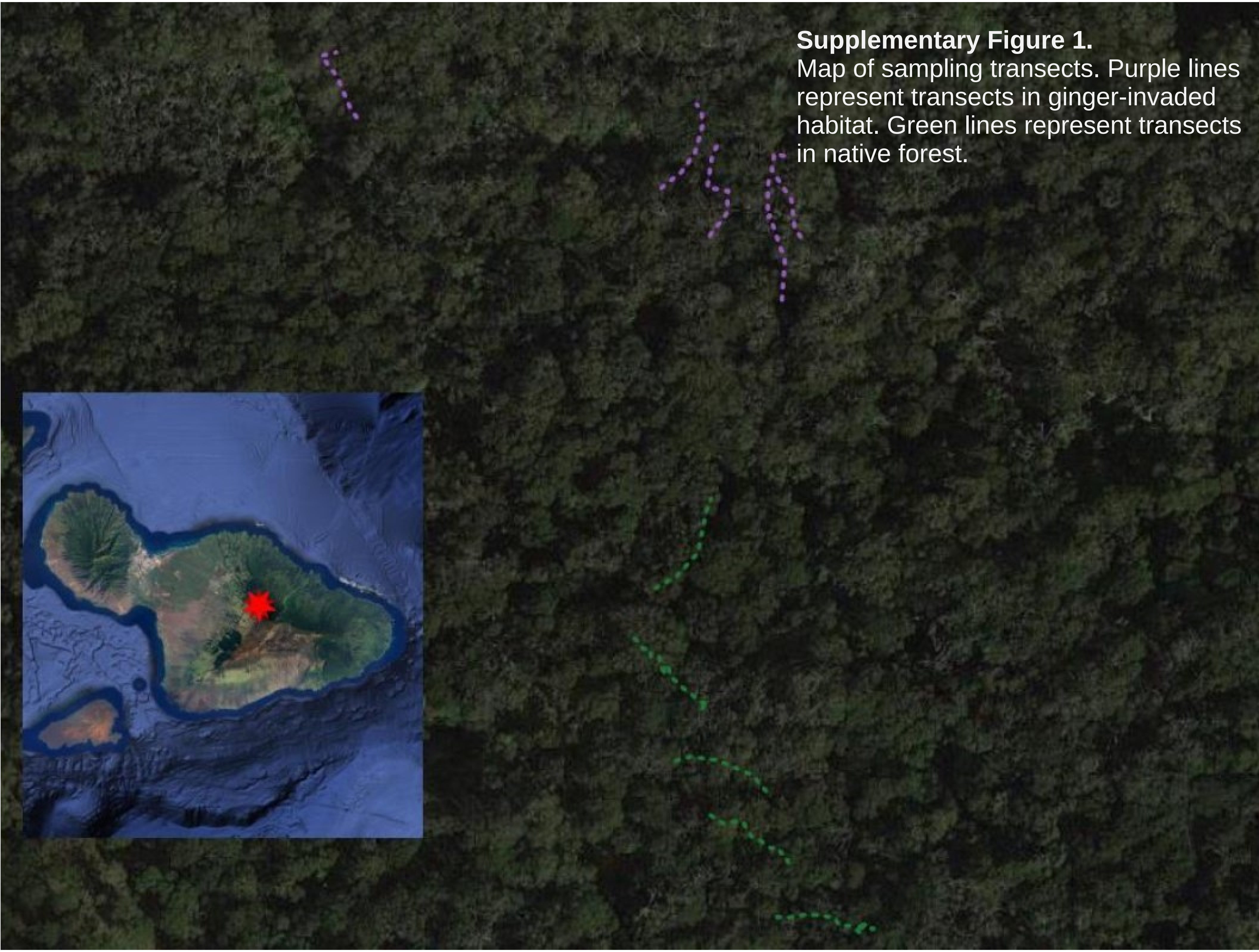

Supplementary Figure 1.
Map of sampling transects. Purple lines represent transects in ginger-invaded habitat. Green lines represent transects in native forest.

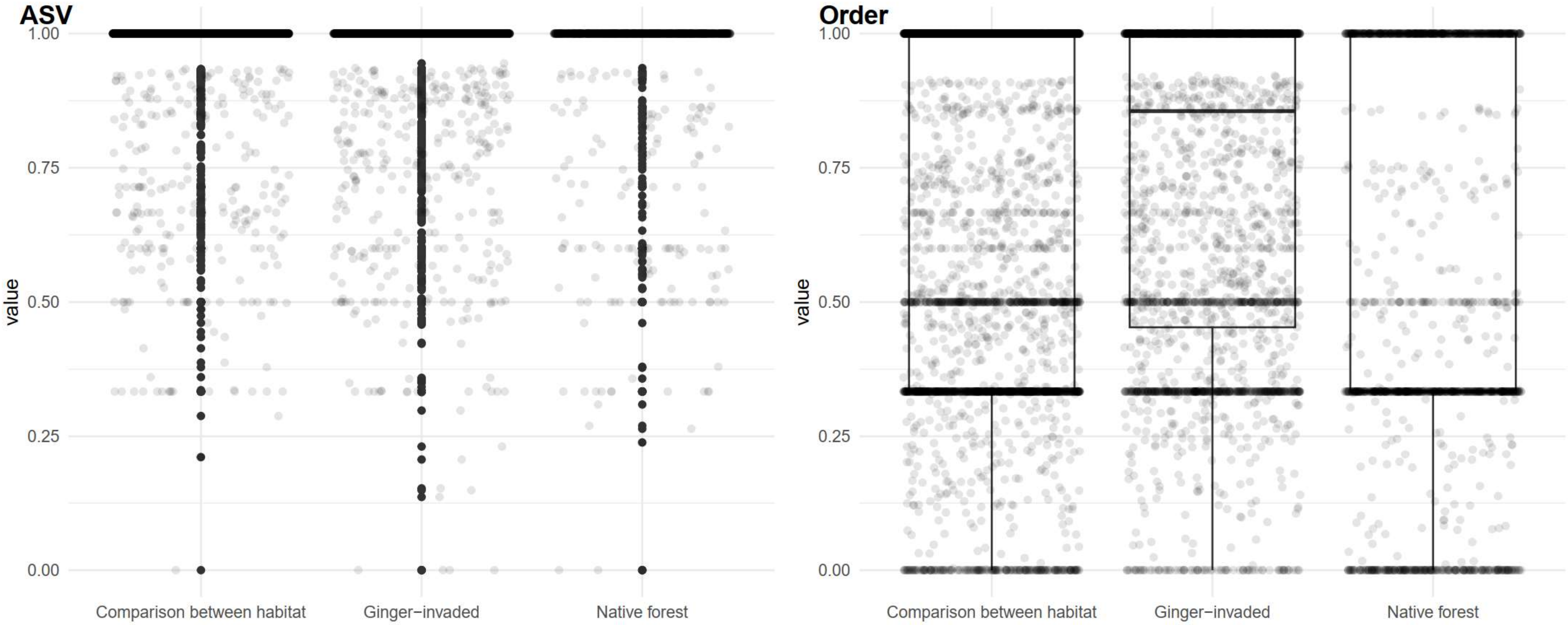

Supplemental Figure 2.
Examples of beta diversity values when calculated between individual spiders. Bands of black on the top are displaying overlapping dots, all with dissimilarity values of 1 (entirely dissimilar)

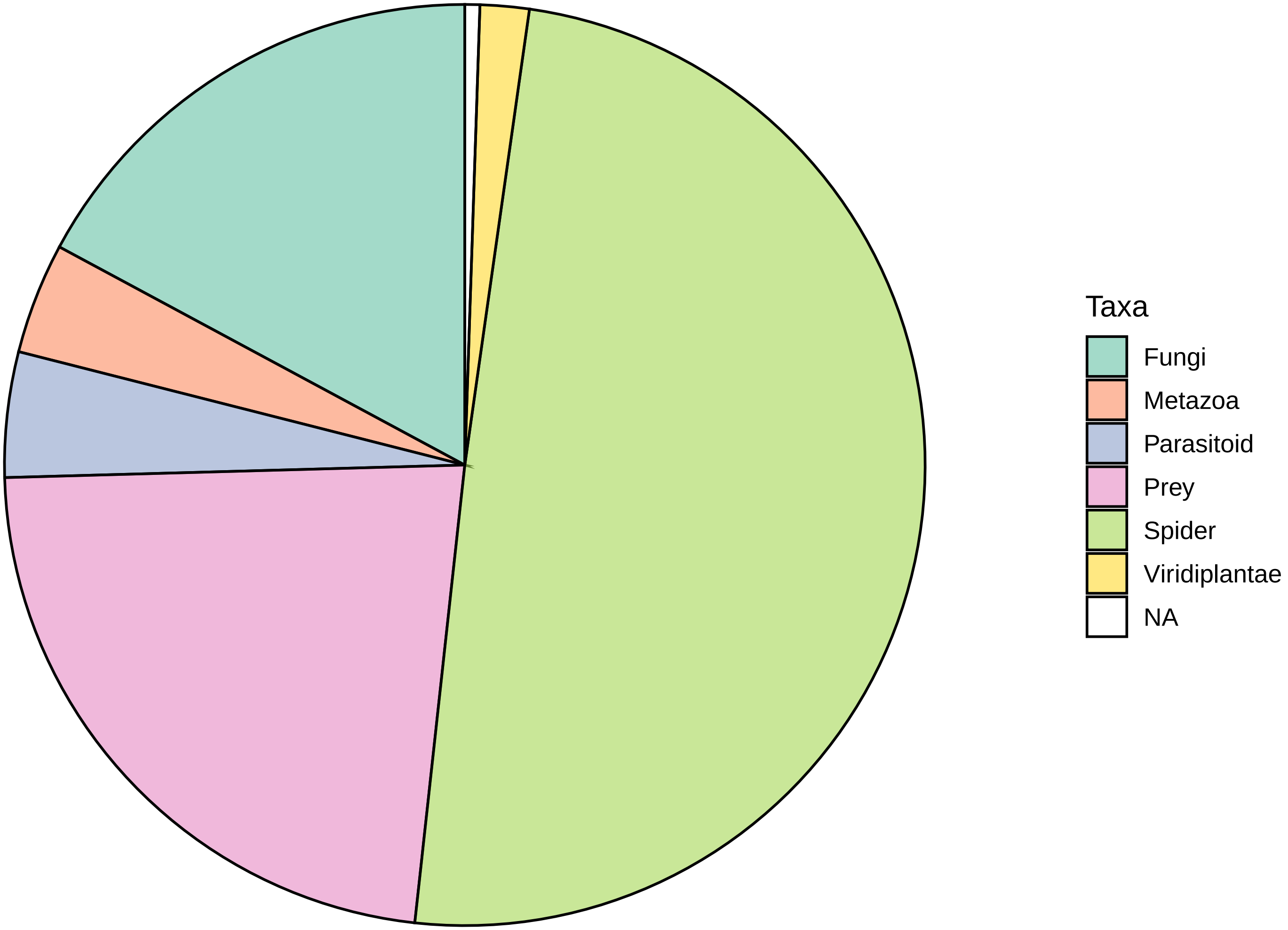

Supplemental Figure 3.
Pie chart displaying the proportion of total reads sequenced by taxonomic group

family	genus	species
**Araneae**		
Ctenidae	Ctenus	
**Coleoptera**		
Ciidae		
Elateridae	Adelocera	
Nitidulidae	Brassicogethes	aeneus
Nitidulidae	Conotelus	
Nitidulidae	Macrostola	
Salpingidae		
Scarabaeidae	Ataenius	
**Diptera**		
Ceratopogonidae		
Dolichopodidae	Campsicnemus	
Dolichopodidae	Eurynogaster	
Drosophilidae		buccata
Drosophilidae		dasycnemia
Drosophilidae		hydei
Drosophilidae		melanogaster
Drosophilidae		petalopeza
Drosophilidae		soonae
Drosophilidae		vulcana
Drosophilidae		
Limoniidae	Limonia	swezeyi
Limoniidae	Limonia	

family	genus	species
Limoniidae		
**Entomobryomorpha**		
Entomobryidae	Homidia	
Paronellidae	Salina	celebensis
Paronellidae	Salina	
Tomoceridae	Tomocerus	
**Hemiptera**		
Aphididae	Myzus	
Cicadellidae	Deltocephalus	
Cicadellidae	Eutettix	
Cicadellidae	Nesophrosyne	cinerea
Cicadellidae	Nesophrosyne	furculata
Cicadellidae	Nesophrosyne	ogradyi
Cicadellidae	Nesophrosyne	
Cicadellidae		
Cixiidae	Oliarus	
Cixiidae		
Delphacidae		
Miridae	Heterotoma	meriopterum
Miridae	Heterotoma	
Miridae	Monalocoris	americanus
Miridae		
**Lepidoptera**		
Bucculatricidae	Ogmograptis	

family	genus	species
Erebidae	Lymantria	monacha
Geometridae	Hydriomena	furcata
Geometridae		
Hesperiidae	Perrotia	
Hesperiidae		
Noctuidae	Noctua	
Notodontidae	Phalera	
Notodontidae		
Nymphalidae		
Sesiidae	Synanthedon	vespiformis
Tortricidae	Eucosma	
**Neuroptera**		
Hemerobiidae	Micromus	
**Psocoptera**		
Trichopsocidae	Trichopsocus	
**Thysanoptera**		
Thripidae	Pezothrips	kellyanus
Thripidae	Taeniothrips	inconsequens

Supplementary Table 1.
Summary of all taxonomic groups identified in prey reads

PERMDISP - ASV Hellinger

Statistic	N	Mean	St. Dev.	Min	Max
Df	2	14.000	18.385	1	27
Sum Sq	2	0.087	0.120	0.002	0.171
Mean Sq	2	0.004	0.003	0.002	0.006
F value	1	0.297		0.297	0.297
Pr(> F)	1	0.590		0.590	0.590

PERMDISP - ASV Incidence

Statistic	N	Mean	St. Dev.	Min	Max
Df	2	14.000	18.385	1	27
Sum Sq	2	0.024	0.034	0.00005	0.049
Mean Sq	2	0.001	0.001	0.00005	0.002
F value	1	0.026		0.026	0.026
Pr(> F)	1	0.873		0.873	0.873

PERMDISP - Order Hellinger

Statistic	N	Mean	St. Dev.	Min	Max
Df	2	14.000	18.385	1	27
Sum Sq	2	0.970	1.199	0.123	1.818
Mean Sq	2	0.095	0.039	0.067	0.123
F value	1	1.821		1.821	1.821
Pr(> F)	1	0.188		0.188	0.188

PERMDISP - Order Incidence

Statistic	N	Mean	St. Dev.	Min	Max
Df	2	14.000	18.385	1	27
Sum Sq	2	0.476	0.649	0.017	0.936
Mean Sq	2	0.026	0.012	0.017	0.035
F value	1	0.494		0.494	0.494
Pr(> F)	1	0.488		0.488	0.488

Supplementary Table 2.
Results from PERMDISP, testing differences in group dispersion.

**Marker**	**Forward** 5`-3`	**Reverse** 5`-3`
16s	ATWACGCTGTTATCCCYAA	ARGACGAGAAGACCCYATA
28s	CCGTCTTGAAACACGGACCA	GWCCTCCATCAGGGTTTCCC
18s	AGCTCTTTCTYGATTCRGTGGGT,<br>GTCTGGTTRATTCCGRTAACGAA	TTGAGCAATAACAGGTCTGTG

Supplementary Table 3.
Sequences of primers used for amplification. Two forward primers were used and combined for amplification of 18s. Primers from Henrik *et al.* 2019.
